# Discontinuation of rLH two days before hCG may increase the number of oocytes retrieved in IVF

**DOI:** 10.1186/1477-7827-8-29

**Published:** 2010-03-23

**Authors:** Jessica B Spencer, Aimee S Browne, Susannah D Copland, Donna R Session

**Affiliations:** 1 Division of Reproductive Endocrinology & Infertility, Department of Gynecology & Obstetrics, Emory University School of Medicine, Atlanta, GA, USA; 2 Division of Reproductive Endocrinology & Infertility, Department of Obstetrics & Gynecology, Duke University School of Medicine, Durham, NC, USA

## Abstract

**Background:**

Administration of recombinant luteinizing hormone (rLH) in controlled ovarian hyperstimulation may benefit a subpopulation of patients. However, late follicular phase administration of high doses of rLH may also reduce the size of the follicular cohort and promote monofollicular development.

**Methods:**

To determine if rLH in late follicular development had a negative impact on follicular growth and oocyte yield, IVF patients in our practice who received rFSH and rLH for the entire stimulation were retrospectively compared with those that had the rLH discontinued at least two days prior to hCG trigger.

**Results:**

The two groups had similar baseline characteristics before stimulation with respect to age, FSH level and antral follicle count. However, the group which had the rLH discontinued at least two days prior to their hCG shot, had a significantly higher number of oocytes retrieved, including a higher number of MII oocytes and number of 2PN embryos.

**Conclusions:**

When using rLH for controlled ovarian hyperstimulation, administering it from the start of stimulation and stopping it in the late follicular phase, at least two days prior to hCG trigger, may increase oocyte and embryo yield.

## Background

The precise benefit and use of recombinant luteinizing hormone (rLH) is still debated. It is unclear which subpopulation benefits the most (e.g. poor responders, extreme pituitary suppression, patients with anovulation). Routine administration of rLH in antagonist cycles may increase the peak serum E2 level but did not increase the number of oocytes retrieved or increase IVF pregnancy rates in one large randomized controlled trial [1]. A recent case-control study however did show a significant increase in implantation rates when rLH was used with antagonist cycles [2]. Adding rLH after day 6 of stimulation in a favorable prognosis population does not seem to improve stimulation or pregnancy rates either [3,4]. However, when an undesirable response occurs after initial stimulation with FSH alone, adding rLH rather than increasing the FSH dose may increase the number of oocytes retrieved [5] and possibly the pregnancy rate per transfer [6]. The optimal dose and timing of rLH in controlled ovarian hyperstimulation is also unclear. One efficacy study showed an increased pregnancy rate with 75 IU vs. 37.5 IU daily (31% vs. 21% respectively) [7].

While the addition of rLH may increase response in COH, very high doses of rLH (660 IU/day) in anovulatory women, who are at particular risk of ovarian hyperstimulation, may promote the growth of a single dominant follicle [8], thereby reducing her risk for ovarian hyperstimulation syndrome. It has therefore been hypothesized that high dose rLH supplementation may reduce the size of the developing follicular cohort. Late follicular phase administration of rLH in doses of 225-450 IU/day has also shown to minimize the number of developing follicles in a smaller earlier study [9].

If poor responders potentially benefit from the addition of rLH but late follicular phase administration reduces the total number of growing midsize follicles, we hypothesized that stopping rLH in late follicular development would increase the number of oocytes obtained at retrieval. We performed a retrospective review of IVF patients in our practice that were stimulated with both rFSH and rLH to determine if stopping the rLH at least two days prior to hCG trigger increased the number of oocytes retrieved.

## Methods

### Study design

Patients who received rLH in an IVF cycle at our clinic between 2005-2007 were identified for chart review after IRB approval. The two groups were assessed for age, maximum FSH level, antral follicle count (AFC), type of stimulation (Lupron microdose co-flare versus Lupron down regulation protocol), whether cancellation occurred for poor response, length of stimulation, daily FSH and rLH dose, and peak estradiol (E2) and progesterone (P4) levels.

Patients on GnRH antagonists were excluded. At our center, a microdose co-flare is preceded by luteal estradiol patches 0.1 mg applied every other day starting on day 20 of the prior cycle. Lupron 20 units BID is then started on day 2 of the cycle, at a concentration of 40 mcg/0.2 mL, and continued until day of hCG. The down regulation protocol is started on day 21 of the preceding cycle, initially at a dose of 20 units a day (1 mg/0.2 mL) until down regulation is achieved. The dose is then lowered to 10 units a day until day of hCG. Patients are generally triggered with urinary hCG 10,000 units when the lead follicle is >20 mm, or two are greater than 18 mm, with estradiol levels at approximately 200 pg/mL per follicle, preferably on day 10 of stimulation.

### Study groups

Both groups started stimulation with both rFSH and rLH. The subjects were divided into two groups: (1) those that had rLH stopped two or more days prior to hCG (with only rFSH for late follicular development), and (2) those that received rFSH and rLH until the day of hCG trigger.

### Outcome variables

Outcomes of interest included the rate of midsize follicular growth (mm/day as measured by ultrasound), number of oocytes retrieved (total and MII), number of 2PNs after fertilization, number of grade 1 embryos available on day 3 of development, the number of embryos available for cryopreservation on day 3, the pregnancy rate as a measure of both positive beta hCG per cycle start and clinical pregnancy by ultrasound per cycle start. The vast majority of our cycles at our center involve a cleavage stage embryo transfer on day 2 or 3, therefore no blastocyst data is available.

### Statistical analysis

The variables were assessed for distribution and considered reasonably normally distributed, therefore continuous variables were compared with a two tailed unpaired t-test, and proportions by Chi-square analysis or in cases of very small numbers, a Fisher's exact test. Statistical analysis was performed with SAS v9.1. A p-value of < 0.05 was considered statistically significant.

## Results

56 patients were included in the study. Table 1 demonstrates that the two groups were similar with respect to age, day 3 FSH level, antral follicle count (AFC), type of stimulation and cancellation rate. These characteristics were also not significant among the patients who proceeded to egg retrieval. While the two groups were similar to each other, they were both in poor prognosis categories: age = 37, FSH > 8 mIU/mL, and AFC < 12, since the majority of good prognosis patients at our clinic receive FSH alone. Consequently, nearly half of the stimulations were with short protocol co-flares, with a significant rate of cancellation (21-28%). The two groups had similar lengths of stimulation, daily FSH dose, and peak serum estradiol and progesterone levels. However, the group which received rLH throughout the entire stimulation (group 2), did have a small but significantly higher daily rLH dose (119 IU/day vs. 97 IU/day, p = 0.038).

**Table 1:** Subgroups baseline characteristics and outcomes

Baseline characteristics	Group 1 (N = 42)	Group 2 (N = 14)	p-val
Age	37.0	37.5	0.718^1^
Max FSH (mIU/mL)	9.32	8.75	0.545^1^
AFC (2-10 mm)	10.98	10.50	0.468^1^
#/% co-flares	22(52.4%)	6(42.9%)	0.537^2^
#/% cancelled	12(28.6%)	3(21.4%)	0.736^3^
**Patients who proceeded to egg retrieval**	**(N = 30)**	**(N = 11)**	**p-val**
Length of stimulation (days)	10.4	11.2	0.166^1^
Daily FSH dose (IU)	395	426	0.381^1^
Daily rLH dose (IU)	97	119	0.038^1^**
Peak E2 (pg/mL)	1892	1629	0.322^1^
Peak P4 (ng/mL)	1.21	0.88	0.099^1^
**Outcomes**	**Group 1**	**Group 2**	**p-val**
Midsize follicular growth (mm/day)	0.96	0.78	0.414^1^
Oocytes retrieved	11.4	7.6	0.041^1^**
Number of MIIs	8.7	4.7	0.019^1^**
Number of 2PNs	6.8	3.5	0.006^1^**
Number of Grade 1 embryos	1.23	1.36	0.817^1^
Embryos for cryopreservation	1.1	0.5	0.450^1^
Positive βhCG/cycle start	27.5%	33.3%	0.726^3^
Clinical pregnancy/cycle start	25.0%	25.0%	1.000^3^

Group 1 (had rLH discontinued at least two days prior to hCG trigger) and Group 2 (had rLH continued throughout stimulation)

^1^Unpaired two tailed t-test, ^2^Chi-sqare analysis, ^3^Fisher's exact test

**Significant assuming α = 0.05

Group 1 (rLH discontinued two or more days prior to hCG trigger) had a significantly higher number of retrieved oocytes (11.4 vs. 7.6, p = 0.041), metaphase II oocytes (MIIs) (8.7 vs 4.7, p = 0.019) and pronuclear stage embryos (2PNs) after one day of culture (6.8 vs 3.5, p = 0.006). Group 1 also had a greater number of embryos for cryopreservation but this was not statistically significant. Clinical pregnancy rates were identical in the two groups.

## Discussion

Discontinuing rLH in late follicular development (at least two days prior to hCG) may increase the number of oocytes, MIIs and 2PNs in poor prognosis patients. While this was demonstrated in this preliminary, small, retrospective study, a larger prospective trial may determine whether this improves pregnancy rates in this subpopulation of IVF patients. It is likely that the use of rLH in an unselected population confers no benefit in live birth rates as demonstrated by a meta analysis involving 701 patients [10]. This was also demonstrated in a recent study of normal responders who received rLH in the early follicular phase [11]. However, it is possible that baseline or stimulation serum LH levels may help determine which group if any benefits from rLH supplementation (and possibly when during the stimulation). One study performed with oocyte donors found rLH was only beneficial in donors with serum LH levels < 1 IU/l at the start of stimulation [12].

## Conclusions

When administering rLH at the beginning of controlled ovarian hyperstimulation for a poor responder, stopping the rLH and continuing FSH stimulation alone at least two days prior to hCG trigger may increase the oocyte yield at egg retrieval, possibly resulting in more MII oocytes and 2PN embryos for the patient.

## Competing interests

JBS, ASB and DRS declare they have no competing interests. SDC serves on the Future Fertility Leaders Advisory Board Meeting for Merck & Co., Inc.

## Authors' contributions

JBS developed study design, analyzed data, statistical methods, and drafted the manuscript. ABS analyzed data, performed statistical analysis and reviewed the manuscript. SDC assembled the raw database, provided critical review and expertise. DRS developed hypothesis, study design and reviewed manuscript. All authors read and approved the final manuscript.

## Authors' information

JBS is an Assistant Professor of Reproductive Endocrinology & Infertility and the Director of the Donor Egg Program at Emory University in Atlanta, GA. ASC is an Assistant Professor of Reproductive Endocrinology & Infertility at Emory University. SDC is an Assistant Professor of Reproductive Endocrinology & Infertility and the director of the Donor Egg and Fertility Preservation Programs at Duke University in Durham, NC. DRS is an Associate Professor and Division Director of Reproductive Endocrinology & Infertility, and the IVF director at Emory University.
